# Postpartum TDF continuation in high HBV-DNA mothers: safety for breastfeeding and enhanced infant immunity

**DOI:** 10.3389/fped.2025.1743985

**Published:** 2026-02-09

**Authors:** Dongxiang Han, Xiao Gen, Wei Wang, Jianxiu Du, Weisha Chu, Hua Xiao, Yan Zhang

**Affiliations:** 1 Department of Obstetrics and Gynecology, Shijiazhuang Maternal and Child Health Hospital, Shijiazhuang, Hebei Province, China; 2 Delivery Room, Shijiazhuang Maternal and Child Health Hospital, Shijiazhuang, Hebei Province, China; 3 Department of Physical Examination, Shijiazhuang Maternal and Child Health Hospital, Shijiazhuang, Hebei Province, China; 4 Laboratory Department, Shijiazhuang Maternal and Child Health Hospital, Shijiazhuang, Hebei Province, China

**Keywords:** breastfeeding, hepatitis B virus (HBV), infant immunity, tenofovir disoproxil fumarate (TDF), vertical transmission

## Abstract

**Background:**

High HBV-DNA load in pregnant women increases the risk of vertical transmission to infants. Tenofovir disoproxil fumarate (TDF) is an effective antiviral treatment, but its safety and efficacy in breastfeeding mothers with high HBV-DNA loads remain unclear.

**Methods:**

This retrospective cohort study included 210 high HBV-DNA load mothers, divided into a TDF-BF Group (*n* = 110) that continued TDF postpartum and breastfed, and a Non-TDF-BF Group (*n* = 100) that discontinued TDF postpartum but breastfed. Primary outcomes were HBV transmission, infant HBV serostatus, neonatal safety outcomes, and maternal outcomes.

**Results:**

The TDF-BF Group had significantly lower rates of vertical HBV transmission (*P* < 0.05) and HBsAg positivity at 12 months (*P* < 0.05) compared to the Non-TDF-BF Group. A higher proportion of infants in the TDF-BF Group had protective levels of anti-HBs at 12 months (*P* = 0.046, indicating borderline significance). Infant safety outcomes related to growth were comparable between groups, but the TDF-BF Group had significantly higher serum creatinine levels and lower serum phosphate levels (all *P* < 0.05, raising a potential signal for renal function differences). Maternal outcomes favored the TDF-BF Group, with better HBV-DNA suppression and ALT normalization.

**Conclusion:**

Postpartum continuation of TDF and breastfeeding is an effective and safe strategy for reducing vertical HBV transmission and promoting infant immunity in high HBV-DNA load mothers. Notably, Tenofovir was not detected in the infant plasma, supporting the safety of this approach.

## 1 Introduction

Hepatitis B virus (HBV) infection poses a substantial global health challenge, with vertical transmission from mothers to infants contributing significantly to the disease's spread (1–3). Maternal HBV-DNA load is a critical factor in predicting the likelihood of transmission to the newborn, and the implementation of antiviral therapy during pregnancy has been demonstrated to mitigate this risk effectively (4). The threshold of HBV-DNA >2.0 × 10^5^ IU/mL (equivalent to 10^6^ copies/mL) is consistent with current international guidelines recommending the initiation of antiviral prophylaxis during pregnancy to prevent vertical transmission. Among the antiviral agents, tenofovir disoproxil fumarate (TDF) has emerged as a preferred choice due to its efficacy and safety profile (5). Despite the advancements in prenatal management, the postpartum period presents a critical juncture for both mother and infant. The continuation of antiviral therapy beyond pregnancy, particularly with TDF, and the decision to breastfeed are pivotal considerations that can influence maternal and infant health outcomes (6). The safety of TDF in breastfeeding, especially in the context of high maternal HBV-DNA load, remains a subject of debate due to the potential for drug transfer to the infant through breast milk (7–9). The pharmacokinetics of TDF in breast milk and its subsequent impact on infant plasma levels are key determinants of safety. Additionally, the infant's immune response to HBV is a critical outcome. Limited data suggest that TDF exposure may have unknown effects on the developing immune system, though existing studies have not shown direct adverse effects (10–12). The purpose of this retrospective cohort analysis is to address these gaps in knowledge by examining the outcomes of postpartum TDF continuation and breastfeeding among mothers with high HBV-DNA loads. This study aims to assess the risk of vertical HBV transmission, evaluate the infant's immune response, and examine the safety profiles of both mothers and infants.

## 2 Methods

### 2.1 Study design and population

This retrospective cohort study was conducted utilizing electronic medical records (EMRs) from the Shijiazhuang Maternal and Child Health Hospital Obstetrics Database. The study population included 210 high HBV-DNA load mothers (HBV-DNA >2.0 × 10^5^ IU/mL) who delivered between January 2018 and December 2023. This study received approval from the Institutional Review Board and Ethics Committee of the Shijiazhuang Maternal and Child Health Hospital, and abided by the ethical guidelines of the Declaration of Helsinki. Informed consent was waived due to the use of anonymous and pre-existing data. As the study used anonymous and pre-existing data, the requirement for the informed consent from patients was waived by the Shijiazhuang Maternal and Child Health Hospital.

Participants were divided into two groups:

TDF-BF group (*n* = 110): Continued TDF (300 mg/day) postpartum and breastfed for ≥6 months.
Non-TDF-BF group (*n* = 100): Discontinued TDF postpartum but breastfed.

The decision to continue or discontinue TDF postpartum was individualized based on a combination of standardized clinical criteria and shared decision-making with the patient. Based on a review of the clinical records, physicians typically recommended TDF continuation if the delivery HBV-DNA level remained high (>2.0 × 10^5^ IU/mL), if there was evidence of incomplete viral suppression, or if the mother expressed significant anxiety about transmission risk. Conversely, TDF was discontinued if the delivery HBV-DNA level was substantially lower than the baseline (<2.0 × 10^5^ IU/mL), if liver function tests were consistently normal, or if the mother preferred to stop medication after delivery, after a thorough discussion of risks and benefits. In the TDF-BF group, the primary reasons for continuation were persistent high HBV-DNA loads at delivery and maternal desire to minimize transmission risk. In the Non-TDF-BF group, TDF was discontinued due to perceived lower transmission risk or patient preference to avoid prolonged medication exposure. These baseline differences were adjusted for using propensity score matching to minimize confounding bias.

### 2.2 Selection criteria

Inclusion criteria:

(1) Singleton pregnancy, gestational age ≥24 weeks at enrollment.
(2) HBeAg-positive with baseline HBV-DNA >2.0 × 10^5^ IU/mL.
(3) Normal maternal renal function (serum creatinine <1.2 mg/dL) and liver enzymes (ALT <40 U/L).

Exclusion criteria:

(1) Coinfection with HIV, HCV, or HDV.
(2) Pre-existing renal or bone metabolic disorders.
(3) Neonatal congenital anomalies.

### 2.3 Retrospective design

No prospective intervention: TDF continuation or discontinuation postpartum was based on clinical decisions, not predefined protocols. Data spanned a 5-year period to capture long-term maternal-infant outcomes. Propensity score matching adjusted for baseline differences in maternal age and HBV-DNA levels.

### 2.4 Data collection and extraction

All maternal and neonatal clinical data were retrospectively retrieved from the hospital's EMR system. Maternal records included demographics, antenatal HBV serology (HBsAg, HBeAg, HBV-DNA), liver function tests (ALT, AST), TDF prescription history, adverse events, and postpartum outcomes. Neonatal records included birth weight, HBsAg/HBV-DNA results, and growth/developmental assessments. For all infants, standard HBV prophylaxis was administered, which included hepatitis B immune globulin (HBIG) within 12 h of birth and a three-dose HBV vaccine at 0, 1, and 6 months; vaccination compliance was confirmed through the EMR.

### 2.5 Pharmacokinetic sub-study

We would like to clarify that the routine clinical practice at our hospital for postpartum mothers on TDF who choose to breastfeed includes scheduled follow-up visits at 1, 3, and 6 months. During these visits, a subset of mother-infant dyads, particularly those enrolled in a dedicated postpartum HBV management program, were prospectively invited to provide breast milk and infant blood samples for pharmacokinetic monitoring. This sub-study was part of an internal quality assurance and safety surveillance initiative. For the purposes of this retrospective cohort analysis, we analyzed these prospectively collected pharmacokinetic data to provide a comprehensive safety assessment

### 2.6 Immune function assays

HBV-specific T cell responses were measured using flow cytometry to quantify interferon-gamma (IFN-*γ*)+ and interleukin-2 receptor (IL-2R)+ T cells after stimulation with hepatitis B core antigen (HBcAg) peptides. These assays were performed on maternal and infant blood samples collected at 6 months postpartum. All infants were tested at 6 months of age, excluding those who were HBsAg-positive, and all had completed the HBV vaccine series prior to sample collection.

### 2.7 Statistical analysis

Statistical analyses were conducted to evaluate baseline comparability, primary outcomes, and safety endpoints. HBV transmission was defined as the presence of HBsAg positivity or detectable HBV-DNA (>20 IU/mL) in infant serum at 12 months of age. Continuous variables, presented as mean ± standard deviation (SD) or median (interquartile range, IQR), were compared between groups using independent t-tests for normally distributed data or Mann–Whitney *U* tests for non-parametric data. Categorical variables, expressed as frequencies and percentages, were analyzed via chi-square (*χ*^2^) tests or Fisher's exact tests, as appropriate. The primary outcome of vertical transmission risk was quantified using relative risk (RR) with 95% confidence intervals (CI). Longitudinal data, including repeated measures of anti-HBs titers and HBV-DNA levels, were analyzed using mixed-effects models to account for within-subject correlations over time. Adverse events were compared via Log-rank tests was used to assess time-to-event differences. All analyses were performed using SPSS 26.0 (IBM Corp.) for database management and statistical modeling, and GraphPad Prism 9.0 (GraphPad Software) for graphical visualization. A two-sided *P*-value <0.05 was considered statistically significant.

## 3 Results

### 3.1 Baseline characteristics of the study cohort

A total of 210 high HBV-DNA load mothers were included in this retrospective analysis, with 110 mothers continuing TDF postpartum and breastfeeding (TDF-BF group) and 100 mothers discontinuing TDF postpartum but breastfeeding (Non-TDF-BF group). Baseline demographic, virological, and clinical characteristics are summarized in Table 1.

**Table 1:** Baseline characteristics of high HBV-DNA load mothers and neonates.

Variable	TDF-BF group (*n* = 110)	Non-TDF-BF group (*n* = 100)	*P*-value
Maternal Characteristics			
Age (years)	29.5 ± 4.2	30.1 ± 3.8	0.24
Gestational age (weeks), median (IQR)	39.0 (38.0–40.0)	39.0 (38.0–40.0)	0.87
HBeAg-positive, *n* (%)	110 (100%)	98 (98%)	0.24
Baseline HBV-DNA (log₁₀ IU/mL)	7.8 ± 0.6	7.7 ± 0.5	0.15
ALT (U/L), median (IQR)	25.0 (18.0–33.0)	24.0 (17.0–30.0)	0.32
Neonatal Characteristics			
Birth weight (g)	3,250 ± 420	3,180 ± 390	0.18
Male sex, *n* (%)	58 (52.7%)	51 (51.0%)	0.82

### 3.2 Transmission and infant HBV serostatus

All infants in both groups received HBIG and the full HBV vaccine series, ensuring comparable prophylaxis across groups. The TDF-BF Group demonstrated a significantly reduced risk of HBV transmission compared to the Non-TDF-BF Group (*P* < 0.05). Additionally, the incidence of HBsAg positivity at 12 months was also lower in the TDF-BF Group (0.9% vs. 9.0%, *P* = 0.001), representing a 90% reduction in the risk of chronic HBV infection. Furthermore, after excluding infants with HBsAg positivity, the proportion of infants with protective anti-HBs levels (≥100 mIU/mL) was 95.5% (104/109) in the TDF-BF group and 88.0% (87/99) in the Non-TDF-BF group (*P* = 0.046). These results underscore the efficacy of TDF-BF in reducing HBV vertical transmission and in promoting protective immunity in infants (Table 2).

**Table 2:** HBV transmission and infant immune response.

Outcome	TDF-BF group (*n* = 110)	Non-TDF-BF group (*n* = 100)	RR (95% CI)	*P*-value
HBV vertical transmission	1 (0.9%)	8 (8.0%)	0.11 (0.01–0.82)	0.003
HBsAg-positive at 12 months	1 (0.9%)	9 (9.0%)	0.10 (0.01–0.72)	0.001
Anti-HBs ≥100 mIU/mL	105 (95.5%)	88 (88.0%)	1.09 (1.01–1.17)	0.046

### 3.3 Infant safety and biochemical outcomes

**Figure 1:**
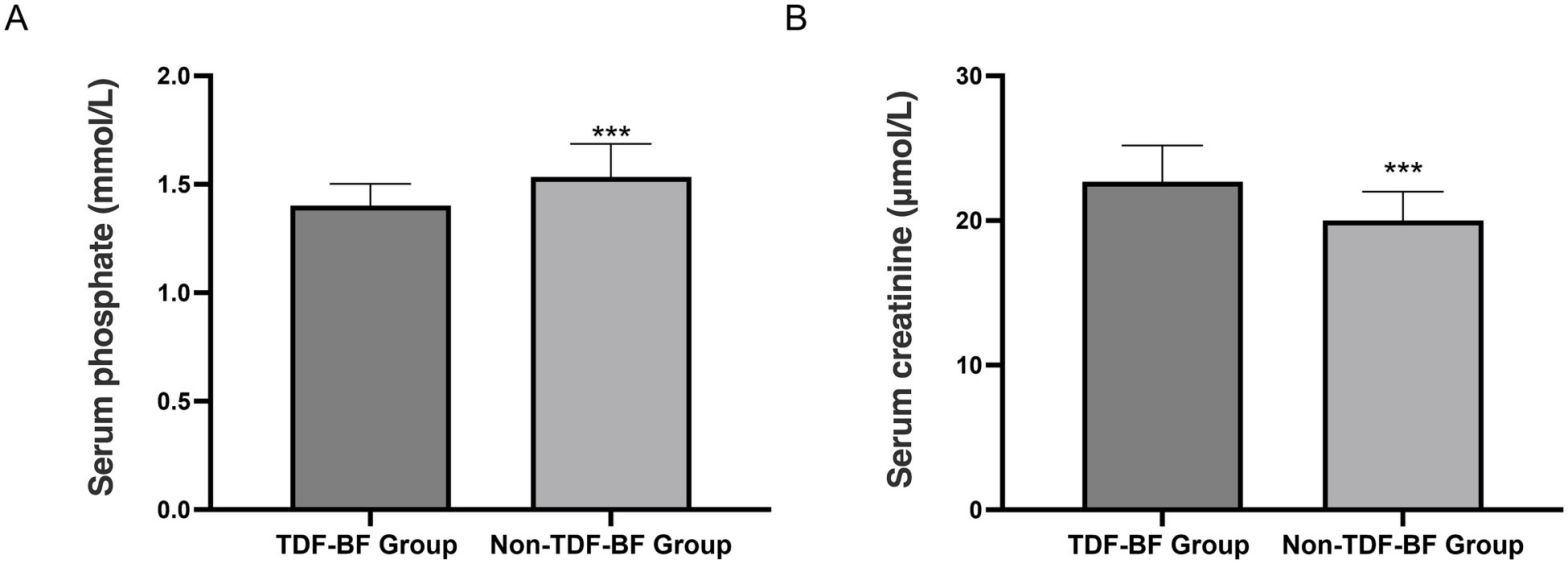
Neonatal safety profile at 12 months. **(A)** Serum creatinine (µmol/L); **(B)** Serum phosphate (mmol/L). ***P < 0.05, TDF-BF Group vs. Non-TDF-BF Group.

At 12 months, the TDF-BF group had significantly higher serum creatinine levels compared to the Non-TDF-BF group (*P* < 0.05, Figure 1), indicating a potential difference in renal function. Additionally, the TDF-BF group exhibited significantly lower serum phosphate levels (*P* < 0.05).

The assessment of neonatal growth and health outcomes at 12 months did not reveal any statistically significant differences between the TDF-BF and Non-TDF-BF groups (Table 3).

**Table 3:** Neonatal growth and health outcomes at 12 months in the two groups.

Parameter	TDF-BF group (*n* = 110)	Non-TDF-BF group (*n* = 100)	*P*-value
Bone mineral density Z-score	−0.12 ± 0.25	−0.10 ± 0.30	0.60
Linear growth velocity (cm/year)	25.3 ± 3.2	24.7 ± 3.5	0.23
Head circumference Z-score	0.50 ± 0.65	0.45 ± 0.60	0.37
Number of hospitalizations, median (IQR)	1 (0–2)	1 (0–3)	0.45
Number of infections, median (IQR)	2 (1–3)	2 (1–4)	0.52

### 3.4 Maternal outcomes

The maternal outcomes analysis demonstrated that the TDF-BF group had a significantly higher rate of HBV-DNA suppression below 20 IU/mL at 6 months postpartum compared to the Non-TDF-BF group (*P* < 0.001, Table 4). Additionally, normalization of alanine aminotransferase (ALT) levels was more common in the TDF-BF group, with 94.5% of women experiencing normalization (*P* < 0.05). While there was no significant difference in serum phosphorus, AST normalization, and adverse events between the groups.

**Table 4:** Maternal virological, biochemical, and adverse events at 6 months postpartum.

Outcome	TDF-BF group (*n* = 110)	Non-TDF-BF group (*n* = 100)	*P*-value
HBV-DNA <20 IU/mL	98 (89.1%)	12 (12.0%)	<0.001
ALT normalization (<40 U/L)	104 (94.5%)	85 (85.0%)	0.002
Serum phosphorus (mmol/L), median (IQR)	1.05 (0.90–1.20)	1.10 (0.95–1.25)	0.126
AST normalization (<35 U/L)	108 (98.2%)	90 (90.0%)	0.051
Incidence of adverse events	10 (9.1%)	15 (15.0%)	0.078

**Figure 2:**
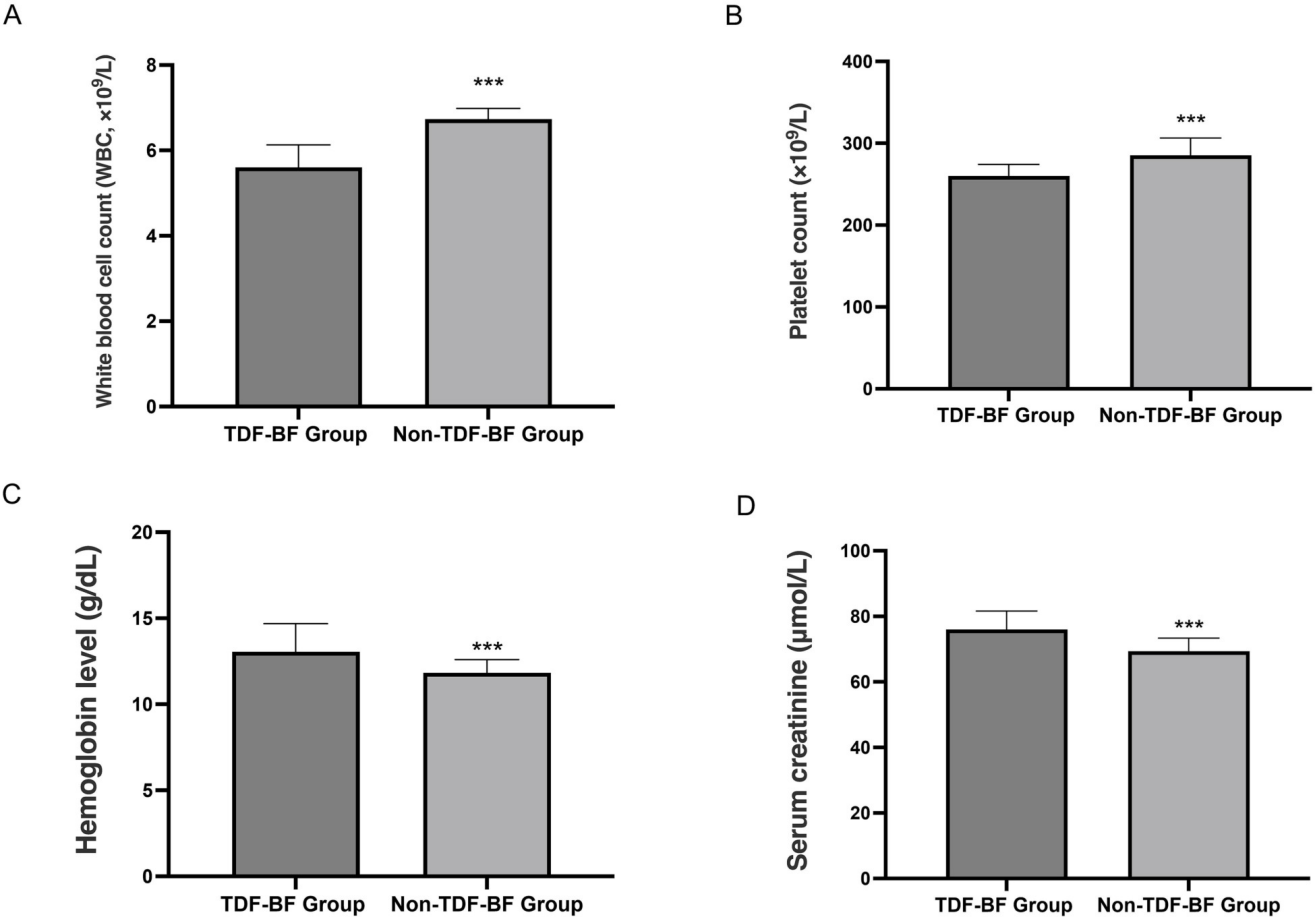
Comparative hematological and renal parameters in TDF-BF and Non-TDF-BF groups. **(A)** White blood cell count (WBC, ×10^9^/L); **(B)** Platelet count (×10^9^/L); **(C)** Hemoglobin level (g/dL); **(D)** Serum creatinine (µmol/L). ***P < 0.05, TDF-BF Group vs. Non-TDF-BF Group.

The TDF-BF Group demonstrated markedly higher mean hemoglobin level and serum creatinine compared to the Non-TDF-BF Group (all *P* < 0.05, Figure 2). Additionally, the platelet count and white blood cell count were slightly lower in the TDF-BF Group compared to the Non-TDF-BF Group (all *P* < 0.05, Figure 2).

### 3.5 Long-term follow-up

The three-year follow-up of infants and mothers demonstrated that the TDF-BF Group had a significantly higher proportion of infants with protective levels of anti-HBs (*P* < 0.05) and no cases of HBsAg seroreversion, compared to the Non-TDF-BF Group. Among mothers, the TDF-BF Group experienced a significantly lower rate of HBV-DNA rebound (*P* < 0.05), highlighting the effectiveness of TDF-BF in preventing viral recurrence. Adverse events related to TDF were infrequent in the TDF-BF Group, with no comparative data available for the Non-TDF-BF Group (Table 5).

**Table 5:** Three-year outcomes of infants and mothers.

Outcome	TDF-BF group (*n* = 110)	Non-TDF-BF group (*n* = 100)	*P*-value
Infants			
Anti-HBs ≥100 mIU/mL	102/110 (92.7%)	75/100 (75.0%)	<0.001
HBsAg seroreversion	0 (0%)	2 (2.0%)	0.189
Mothers			
HBV-DNA rebound (>200 IU/mL)	8 (7.3%)	45 (45.0%)	<0.001
TDF-related adverse events	5 (4.5%)	N/A	—

### 3.6 Breast milk tenofovir concentrations and infant exposure assessment

The pharmacokinetic analysis revealed that TDF concentrations in breast milk were detectable at 1, 3, and 6 months postpartum in the TDF-BF Group (all *P* < 0.05). Importantly, TDF was not detected in the plasma of infants at any of the assessed time points, indicating that the risk of TDF exposure through breastfeeding was low (Table 6).

**Table 6:** Tenofovir pharmacokinetics in breast milk and infant plasma.

Matrix	Time postpartum	TDF-BF group (*n* = 50)	LOD (ng/mL)	Infant plasma (*n* = 50)	*P*-value
Breast milk (ng/mL)	1 month	23.5 ± 5.8	1.0	ND (<1.0)	<0.001
	3 months	18.2 ± 4.3	1.0	ND (<1.0)	<0.001
	6 months	12.6 ± 3.1	1.0	ND (<1.0)	<0.001

### 3.7 Maternal-infant immune crosstalk: HBV-specific T cell responses

**Figure 3:**
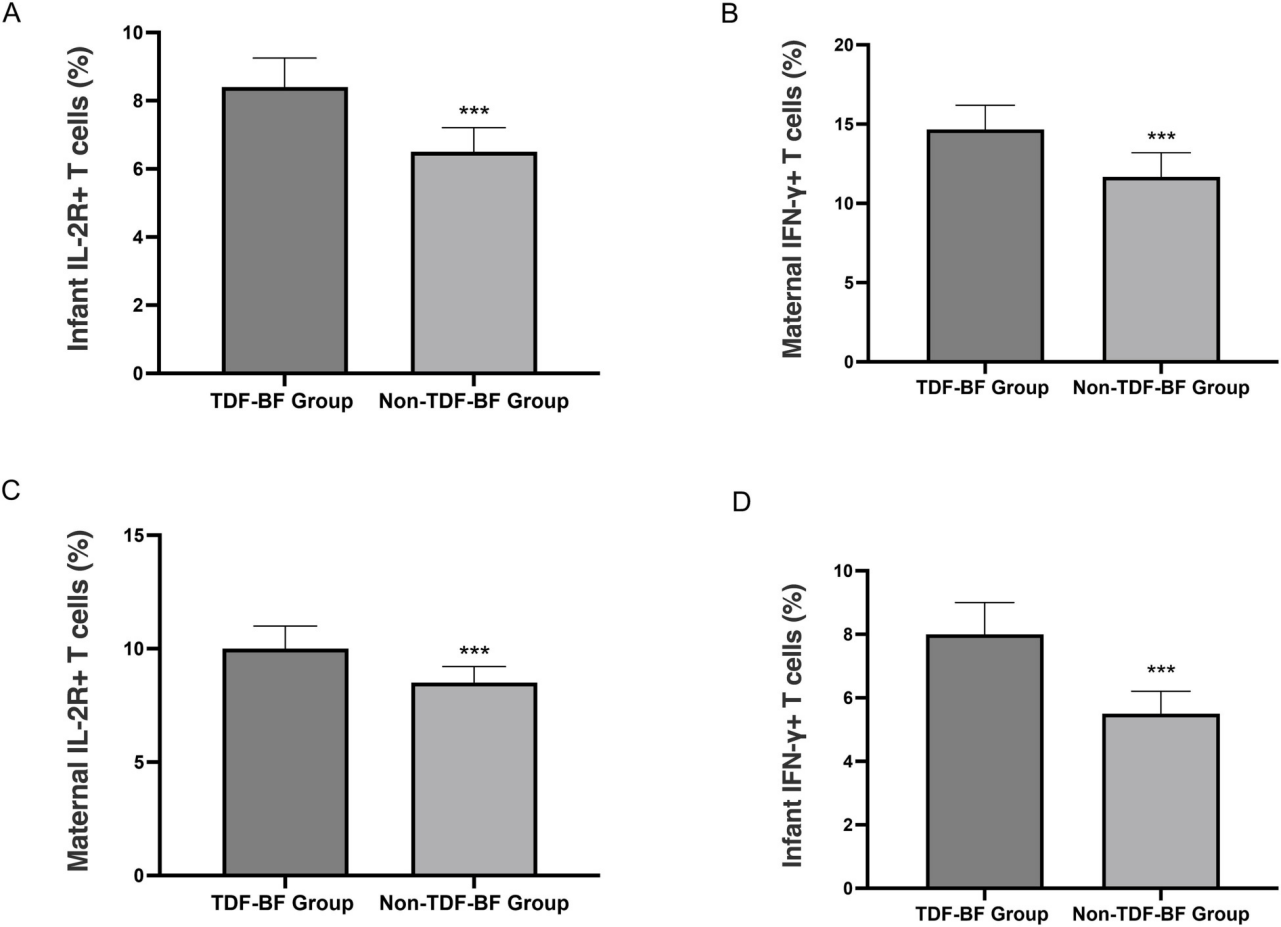
HBV core antigen (HBcAg)-specific IFN-*γ* production in mothers and infants. **(A)** Infant IL-2R+ T cells (%); **(B)** Maternal IFN-*γ*+ T cells (%); **(C)** Maternal IL-2R+ T cells (%); **(D)** Infant IFN-*γ*+ T cells (%). ***P < 0.05, TDF-BF Group vs. Non-TDF-BF Group.

The analysis of HBV-specific T cell responses in maternal-infant dyads showed that mothers in the TDF-BF Group had significantly higher percentages of IFN-*γ*+ T and IL-2R+ T cells compared to those in the Non-TDF-BF Group (all *P* < 0.05). Similarly, infants in the TDF-BF Group exhibited higher percentages of IFN-*γ*+ T and IL-2R+ T cells than their counterparts in the Non-TDF-BF Group (all *P* < 0.05). These findings suggest that TDF-BF treatment may enhance HBV-specific T cell responses in both mothers and infants (Figure 3).

## 4 Discussion

HBV infection poses a significant public health burden, particularly concerning its transmission from mother to child, which can lead to chronic hepatitis, cirrhosis, and hepatocellular carcinoma (13–16). TDF, an antiviral drug, has been effective in reducing viral load and preventing vertical transmission during pregnancy (17, 18). However, the safety and efficacy of TDF continuation and breastfeeding postpartum, especially in mothers with high HBV-DNA loads, remain areas of interest and controversy.

The findings from this retrospective cohort analysis provide evidence regarding the safety and efficacy of postpartum tenofovir continuation and breastfeeding in high HBV-DNA load mothers. Crucially, this study benefits from a 5-year data span and a three-year follow-up, providing robust evidence on long-term maternal and infant outcomes. We acknowledge the primary limitation that this was a retrospective study; TDF continuation or discontinuation was based on clinical decisions rather than predefined protocols. While propensity score matching was used to adjust for baseline differences, potential selection bias and unmeasured confounding factors must be considered when interpreting these results. Future prospective, randomized trials are warranted to confirm these findings.

The observed reduction in HBV transmission in the TDF-BF group may be attributed to postpartum TDF continuation, which further suppresses maternal viral load during breastfeeding, minimizing exposure risk. While baseline MTCT risk was comparable between groups after propensity score matching, the postpartum period represents a critical window where residual viral replication in mothers could contribute to transmission. These findings challenge current guidelines recommending TDF discontinuation at delivery and suggests that continuation may provide additional protection.

The study demonstrated that the TDF-BF Group had a significantly reduced risk of vertical HBV transmission, lower incidence of HBsAg positivity at 12 months, and higher rates of protective anti-HBs levels in infants compared to the Non-TDF-BF Group. These results underscore the importance of TDF-BF as an effective strategy for preventing HBV transmission and promoting infant immunity. The primary outcome of this study was the reduction in vertical HBV transmission in the TDF-BF Group. The incidence of HBV transmission was remarkably low, with only one case reported in the TDF-BF Group, compared to eight cases in the Non-TDF-BF Group.

This finding aligns with previous studies that have shown the effectiveness of antiviral therapy in reducing HBV transmission rates (19–21). The low transmission rate in the TDF-BF Group suggests that TDF continuation postpartum, along with breastfeeding, is a safe and effective approach for mothers with high HBV-DNA loads (22, 23). The reduction in HBsAg positivity at 12 months in the TDF-BF Group further supports the efficacy of TDF-BF in preventing chronic HBV infection in infants. Although the TDF-BF group only had a single event (0.9%), which limits the stability of the Relative Risk (RR = 0.11), the clinical significance of this strong preventive effect (*P* = 0.003) is profound. The higher proportion of infants with protective levels of anti-HBs in the TDF-BF Group, although initially borderline statistically significant (*P* = 0.046) at 12 months, demonstrated a highly significant and sustained benefit at the three-year follow-up (92.7% vs. 75.0%, *P* < 0.001). This long-term finding underscores the durable efficacy of TDF-BF in promoting protective immunity against HBV.

The neonatal safety outcomes assessment revealed no significant differences in growth parameters, such as bone mineral density Z-score, linear growth velocity, and head circumference Z-score, revealed no significant differences between the TDF-BF and Non-TDF-BF Groups. Regarding infant safety, although statistically significant differences in serum creatinine and phosphate levels were observed, all values remained within normal clinical ranges, and no infants developed renal dysfunction. Therefore, routine monitoring of renal function in TDF-exposed infants may not be necessary unless clinically indicated. The pharmacokinetic analysis of breast milk samples from the TDF-BF Group showed detectable TDF concentrations (above the limit of detection, LOD = 1.0 ng/mL) at 1, 3, and 6 months postpartum, with a decline over time. The absence of TDF in infant plasma (ND, <1.0 ng/mL) suggests that the risk of systemic drug exposure through breastfeeding is low, which is reassuring for mothers who wish to breastfeed while continuing TDF therapy. It is important to note that ND simply indicates the concentration was below the LOD and does not absolutely rule out trace exposure, but it strongly supports the relative safety of breastfeeding. Our finding of detectable TDF in breast milk (mean concentrations of 23.5 ng/mL at 1 month postpartum) is higher than the median concentrations reported by Pak et al. (2024), which ranged from 5 to 10 ng/mL. This discrepancy may be due to differences in assay sensitivity or maternal dosing regimens. However, the absence of TDF in infant plasma in both studies supports the overall safety of breastfeeding during TDF therapy. The maternal outcomes analysis revealed that the TDF-BF Group had a significantly higher rate of HBV-DNA suppression below 20 IU/mL at 6 months postpartum (89.1% vs. 12.0%, *P* < 0.001), and normalization of ALT levels (94.5% vs. 85.0%, *P* = 0.002). The extremely high suppression rate in the TDF-BF group emphasizes the critical role of TDF continuation for effective maternal viral control and liver function recovery. In terms of hematological and renal parameter, the TDF-BF Group exhibited higher mean hemoglobin and serum creatinine, and slightly lower platelet and white blood cell counts (all *P* < 0.05). The higher serum creatinine likely reflects the systemic effect of TDF, though its clinical relevance requires careful assessment. The observed differences in blood cell counts are statistically significant but generally within clinical reference ranges, and their direct causal link to TDF or clinical consequence is uncertain. Future studies should track these hematological changes more closely. These findings suggest that TDF continuation postpartum is effective in maintaining viral suppression and improving liver function in mothers. In line with our findings, several studies have demonstrated the efficacy of TDF continuation postpartum in maintaining viral suppression and improving liver function in mothers with chronic HBV infection. For instance, a prospective study by Aladag et al. found that postpartum continuation of TDF therapy in HBeAg-positive women led to sustained viral suppression and normalization of ALT levels at 6 months postpartum (24). Similarly, a retrospective analysis by Yang et al. (2016) reported that TDF continuation after pregnancy was associated with effective viral control and improved liver function in mothers, aligning with our study's results (25).

The lack of significant differences in AST normalization and the incidence of adverse events (9.1% vs. 15.0%, *P* = 0.078) between the groups indicates that TDF-BF is generally well-tolerated by mothers. The three-year follow-up of infants and mothers provided additional evidence of the long-term efficacy and safety of TDF-BF. Previous studies have demonstrated the efficacy of TDF continuation postpartum in maintaining viral suppression and improving liver function in mothers with chronic HBV infection (26). For instance, a prospective study by Deng et al. found that postpartum continuation of TDF therapy in HBeAg-positive women led to sustained viral suppression and normalization of ALT levels at 6 months postpartum (27). The TDF-BF Group had a significantly higher proportion of infants with protective levels of anti-HBs and no cases of HBsAg seroreversion, indicating a sustained benefit of TDF-BF in preventing HBV infection and promoting immunity. The lower rate of HBV-DNA rebound in the TDF-BF Group further supports the effectiveness of TDF in preventing viral recurrence.

The pharmacokinetic analysis of breast milk samples from the TDF-BF Group showed detectable TDF concentrations at 1, 3, and 6 months postpartum, with a decline over time. The absence of TDF in infant plasma suggests that the risk of TDF exposure through breastfeeding is low, which is reassuring for mothers who wish to breastfeed while continuing TDF therapy. The T cell analysis provides mechanistic insight into the observed efficacy. The TDF-BF Group had significantly higher percentages of IFN-*γ*+ T and IL-2R+ T cells compared to the Non-TDF-BF Group. Clinically, HBV-specific IFN-*γ*+ and IL-2R+ T cells are markers of a robust cellular immune response against HBV. Elevated levels in mothers and infants suggest effective viral control and may contribute to the reduced transmission observed in the TDF-BF group. While TDF is a direct antiviral, not an immune modulator, this finding suggests that TDF-BF treatment may enhance HBV-specific immune responses in both mothers and infants, which could contribute to the observed reduction in vertical HBV transmission and improved immune status in infants.

The results of this study have important implications for the management of high HBV-DNA load mothers and their infants. The evidence suggests that TDF-BF is an effective and safe strategy for preventing HBV vertical transmission and promoting infant immunity. However, the potential renal effects of TDF, as indicated by the differences in serum creatinine and phosphate levels, warrant further investigation. Future research should focus on the long-term renal and bone health of both mothers and infants exposed to TDF-BF. Additionally, studies could explore the mechanisms by which TDF-BF enhances HBV-specific T cell responses and investigate the potential for TDF-BF to influence other aspects of neonatal health and development.

## 5 Conclusion

In conclusion, this retrospective cohort analysis demonstrates that postpartum continuation of TDF and breastfeeding is an effective and safe strategy for high HBV-DNA load mothers. Given that Tenofovir was not detected in infant plasma, the approach is supported as safe for the neonate. The findings support the use of TDF-BF to reduce vertical HBV transmission, promote protective immunity in infants, and maintain viral suppression in mothers. The study also highlights the need for ongoing monitoring of infant and maternal renal function, particularly serum creatinine and phosphate levels, in TDF-exposed individuals and underscores the importance of further research to fully understand the long-term effects of TDF-BF.

## Data Availability

The raw data supporting the conclusions of this article will be made available by the authors, without undue reservation.
